# Impairment of Lhca4, a subunit of LHCI, causes high accumulation of chlorophyll and the stay-green phenotype in rice

**DOI:** 10.1093/jxb/erx468

**Published:** 2018-01-03

**Authors:** Hiroshi Yamatani, Kaori Kohzuma, Michiharu Nakano, Tsuneaki Takami, Yusuke Kato, Yoriko Hayashi, Yuki Monden, Yutaka Okumoto, Tomoko Abe, Toshihiro Kumamaru, Ayumi Tanaka, Wataru Sakamoto, Makoto Kusaba

**Affiliations:** 1 Graduate School of Science, Hiroshima University, Higashi-Hiroshima, Hiroshima, Japan; 2 Institute of Plant Science and Resources, Okayama University, Kurashiki, Okayama, Japan; 3 Nishina Center for Accelerator-Based Science, RIKEN, Wako, Saitama, Japan; 4 Graduate School of Agriculture, Kyoto University, Kyoto, Kyoto, Japan; 5 Faculty of Agriculture, Kyushu University, Fukuoka, Japan; 6 Institute of Low Temperature Science, Hokkaido University, Sapporo, Japan

**Keywords:** Chlorophyll, Lhca4, light-harvesting complex, long-term acclimation, rice, state transition, stay-green

## Abstract

Chlorophyll is an essential molecule for acquiring light energy during photosynthesis. Mutations that result in chlorophyll retention during leaf senescence are called ‘stay-green’ mutants. One of the several types of stay-green mutants, Type E, accumulates high levels of chlorophyll in the pre-senescent leaves, resulting in delayed yellowing. We isolated *delayed yellowing1-1* (*dye1-1*), a rice mutant whose yellowing is delayed in the field. *dye1-1* accumulated more chlorophyll than the wild-type in the pre-senescent and senescent leaves, but did not retain leaf functionality in the ‘senescent green leaves’, suggesting that *dye1-1* is a Type E stay-green mutant. Positional cloning revealed that *DYE1* encodes Lhca4, a subunit of the light-harvesting complex I (LHCI). In *dye1-1*, amino acid substitution occurs at the location of a highly conserved amino acid residue involved in pigment binding; indeed, a severely impaired structure of the PSI-LHCI super-complex in *dye1-1* was observed in a blue native PAGE analysis. Nevertheless, the biomass and carbon assimilation rate of *dye1-1* were comparable to those in the wild-type. Interestingly, Lhcb1, a trimeric LHCII protein, was highly accumulated in *dye1-1*, in the chlorophyll–protein complexes. The high accumulation of LHCII in the LHCI mutant *dye1* suggests a novel functional interaction between LHCI and LHCII.

## Teaser

Impairment of Lhca4, a subunit of LHCI, causes high chlorophyll content and LHCII accumulation in rice, suggesting a novel functional interaction between LHCI and LHCII.

## Introduction

Chlorophyll synthesis and breakdown are strictly regulated in plants not only because it is an essential photosynthetic molecule, but also because in its free form it photo-oxidatively damages cells (Tanaka *et al.*, 2011). Mutants that retain the greenness of leaves under senescence-inducing conditions are called stay-green mutants, and they are classified into two types: functional and non-functional. Functional stay-green mutants retain the green of leaves via delayed senescence, whereas the greenness of leaves is not necessarily correlated with leaf functionality in the non-functional mutants. The non-functional stay-green mutants can be further classified (Thomas and Howarth, 2000). The Type C mutants show impairment in chlorophyll degradation, with the majority of the mutations being in the genes that encode chlorophyll-degrading enzymes, such as the Chl*a*-degrading enzyme SGR/NYE1, Chl*b*-degrading enzymes NYC1 and NOL, and pheophytinase PPH/NYC3 (Ren *et al.*, 2007; Kusaba *et al.*, 2007; Park, 2007; Morita *et al.*, 2009; Sato *et al.*, 2009; Schelbert *et al.*, 2009; Shimoda *et al.*, 2016). In addition, mutants of a chloroplast protein THF1/NYC4 and a small subunit of the PSII core complex, PsbM/CytG, show the stay-green phenotype via inhibition of the degradation of chlorophyll–protein complexes (Huang *et al.*, 2013; Yamatani *et al.*1, 2013; Kohzuma *et al.*, 2017). Although the genes associated with these mutations do not encode chlorophyll-degrading enzymes, the mutants are classified as Type C because chlorophyll degradation is impaired during senescence. Another class of non-functional stay-green mutants, Type E, accumulates high levels of chlorophyll in the pre-senescent leaves, resulting in longer retention of greenness than in those of the wild-type. In other words, Type E can be designated as a mutant with impairments in the proper regulation of chlorophyll accumulation.

Chlorophyll is contained in the photosystems in the thylakoid membranes. PSII uses light energy for the extraction of electrons from water, resulting in the production of oxygen. The photo-excited electrons are transferred to PSI through the cytochrome *b*_*6*_*f* complex and are used to produce NADPH. The proton gradient formed during the electron transfer process is used for the production of ATP. The light-harvesting complex I (LHCI) is an antenna complex for PSI, and consists of four subunits, Lhca1–Lhca4, in algae and land plants. Lhca1/Lhca4 and Lhca2/Lhca3, respectively, form dimers. The light-harvesting complex II (LHCII) is an antenna complex for PSII, encoded by *Lhcb1*–*Lhcb6*. Lhcb1, 2, and 3 are the major LHCII subunits and form trimers. Lhcb4, 5, and 6 are minor LHCII subunits that exist as monomers. Higher plants contain Chl*a* and Chl*b*. Chl*a* is a major chlorophyll found in all chlorophyll–protein complexes, whereas Chl*b* is found only in the light-harvesting complexes, LHCI and LHCII.

For optimal photosynthesis, the performances of PSI and PSII should be balanced (Minagawa, 2011). Photosynthetic organisms have several mechanisms to tune the performances of PSI and PSII. State transition is one such mechanism, wherein trimeric LHCII moves to PSI via phosphorylation by STN7 kinase, and functions as an antenna of PSI under ‘PSII-excess’ light conditions (Depège *et al.*, 2003; Bellafiore *et al.*, 2005). This condition is called State 2, while State 1 is induced by a ‘PSI-excess’ situation. State transition is a short-term acclimation under changing light conditions. In addition, plants perform long-term acclimation as well. For example, under lengthy PSII-excess conditions, Arabidopsis increases PSI by increasing the *psaA* and *psaB* mRNA levels (Pesaresi *et al.*, 2009).

LHCII is typically considered an antenna of PSII, except under specific conditions, such as State 2. However, accumulating evidence suggests that LHCII acts as an antenna of PSI even under moderate light conditions (Wientjes, *et al.*, 2013; Grieco *et al.*, 2015). LHCII could be an efficient antenna for PSI, and trimeric LHCII interacts with PSI even in State 1, where LHCII phosphorylation is absent (Benson *et al.*, 2015; Grieco *et al.*, 2015).

In this study, we isolated a novel rice mutant that retains green of leaves in the field in autumn. This stay-green mutant, *delayed yellowing1* (*dye1*), accumulates a higher amount of chlorophyll than the wild-type in the pre-senescent leaves, suggesting that it is a Type E mutant. To our knowledge, this is the first empirical report on Type E stay-green mutants. Positional cloning revealed that *DYE1* encodes Lhca4, a subunit of LHCI. Recent reports using *Lhca* mutants in Arabidopsis have revealed various functions of LHCI in addition to its role as an antenna of PSI, such as its involvement in state transition (Benson *et al.*, 2015; Bressan *et al.*, 2016). Interestingly, Lhcb1 was highly accumulated among the chlorophyll–protein complexes in the pre-senescent *dye1-1* leaves, suggesting that there is a novel functional interaction between LHCI and LHCII.

## Materials and methods

### Plant material

*dye1-1* was isolated from a rice M_2_ population (*Oryza sativa* L. ‘Nipponbare’) irradiated with carbon ion beams (1.6 GeV). *dye1-2* was isolated from the N-methyl-N-nitrosourea-mutagenized Nipponbare pool by using TILLING-based screening (Suzuki *et al.*, 2008). The plants were cultivated in pots under field conditions.

### Photosynthetic parameters

Foliar chlorophyll content was measured non-destructively using a SPAD-502 Plus instrument (KONICA MINOLTA; http://www.konicaminolta.jp, last accessed 24 December 2017). For pigment extraction, leaves were ground in a mortar in liquid nitrogen and extracted using 80% acetone. Chl*a* and Chl*b* levels were determined as described by Porra *et al.* (1989). Maximum quantum yield (*F*_v_/*F*_m_) and the carbon fixation rate were measured using a LI-6400XT portable photosynthesis system (LI-COR; http://www.licor.com/, last accessed 24 December 2017) at 30 °C, with a CO_2_ concentration of 400 ppm, humidity of 70–80%, and photon flux density of 2000 μmol m^–2^ s^–1^. Oxidation of P700 was measured using a Dual-PAM-100 chlorophyll fluorescence and P700 photosynthesis analyser (Walz; http://www.walz.com/, last accessed 24 December 2017). The antenna function of LHCI was estimated as the time required to reach two-thirds of the maximum P700 oxidation with far-red light (intensity 2). State transition (*F*_r_) was determined as described by Lunde *et al.* (2000) using a JUNIORPAM fluorometer (Walz). *F*_r_ is calculated using the following formula: *F*_r_=[(*F*_i_′–*F*_i_)–(*F*_ii_′– *F*_ii_)]/(*F*_i_′–*F*_i_). *F*_i_ and *F*_ii_ are the fluorescence in the presence of PSI light in States 1 and 2, respectively, whereas *F*_i_′ and *F*_ii_′ are the fluorescence in the absence of PSI light in State 1 and 2, respectively.

### Quantitative RT-PCR

Total RNA was extracted from the leaves of the wild-type and *dye1-1* plants using a total RNA extraction kit (RBC Bioscience; http://www.rbcbioscience.com/, last accessed 24 December 2017). First-strand cDNA was synthesized from 500 ng total RNA using ReverTra ACE qPCR RT Master Mix with gRNA Remover (TOYOBO; http://www.toyobo.co.jp/, last accessed 24 December 2017). The transcript level was determined by quantitative RT-PCR using a KAPA SYBR FAST qPCR kit (KAPA Biosystems; http://www.kapabiosystems.com/, last accessed 24 December 2017) and a Rotor-Gene Q real-time PCR cycler (Qiagen; http://www.qiagen.com/, last accessed 24 December 2017). The primers used for amplification are listed in Supplementary Table S1 at *JXB* online.

### Protein analysis

Total protein was extracted from a 100-mg (FW) sample of leaves from both the wild-type and *dye1-1*, using 400 µl of 2×SDS buffer [0.125 M Tris, pH 6.8, 4% SDS, 4% mercaptoethanol, 1% bromophenol blue (BPB), 20% glycerol]. The extracted proteins were diluted to one-fifth concentration using 1×SDS buffer (62.5 mM Tris, pH 6.8, 2% SDS, 2% mercaptoethanol, 0.5% BPB, 10% glycerol) and subjected to SDS-PAGE with or without boiling. The antibodies against Lhca1–Lhca4, and Lhcb1 for western blot analysis were purchased from Agrisera (http://www.agrisera.com/en/info/home.html, last accessed 24 December 2017), while the anti-PsaF antibody was provided by Y. Takahashi (Graduate School of Natural Science and Technology, Okayama University, Japan). The antibody against D1 was described previously by Kato *et al.* (2012). Detection of each protein was performed using an ECL Prime western blotting detection system (GE Healthcare; http://www3.gehealthcare.com/, last accessed 24 December 2017) and an ODYSSEY Fc imaging system (LI-COR). Quantification of band intensity in the western blot analysis was performed using Image Studio Ver 5.2 (LI-COR). An SDS-PAGE gel was stained by Coomassie Brilliant Blue R-250 to detect the Rubisco large subunit. Blue native PAGE analysis was performed using thylakoids solubilized in 1% β-dodecyl-maltoside, as described by Yamatani *et al.* (2013).

### Positional cloning

Twenty-two stay-green F_2_ segregants from a cross between *dye1-1* and the *japonica* rice Gimbozu EG4 were used for coarse-mapping. For fine-mapping, about 3000 F_2_ plants from a cross between *dye1-1* and a line from the chromosome segment substitution line (CSSL) between Koshihikari and Kasalath (SL224) were used (Ebitani *et al.*, 2005). The DNA markers used in positional cloning are described in Supplementary Table S2.

### Whole-genome sequencing

Whole-genome sequencing of *dye1-1* was performed with HiSeq2000 (Illumina; https://www.illumina.com/, last accessed 24 December 2017). Three candidate mutations against the Nipponbare genome sequence with quality over 50 were detected within the 43.1-kb *DYE1* candidate region. Among the three candidate mutations, the G-to-A substitution in the second exon of *Lhca4* was the only ‘homozygous’ mutation.

### Transformation experiments

For complementation analysis, the 6-kb genomic fragment that contains the entire Os08g0435900 gene was amplified by PCR using Prime STAR GXL polymerase (TaKaRa; http://www.takara-bio.com/, last accessed 24 December 2017) and the primers DYE1 F1 (5′-TAGGCGCGCCAAGCTTA TGCAGTATGCTGTGACGGT-3′) and DYE1 R1 (5′-TTAATTAAGAATT CGAGCTCCACGCGAGG CCGCGAGAGGG-3′). Amplified DNA was cloned into the HindIII-SacI site of pZH2B, a binary vector derived from pPZP202 (Hajdukiewicz *et al.*, 1994), using the In-fusion HD cloning kit (TaKaRa). *dye1-1* calli were transformed with this construct by *Agrobacterium*-mediated transformation, as described by Fukuoka *et al.* (2000).

### Accession numbers

The following rice genes were used in the analysis: *NYC3* (Os06g0354700), *SGR* (Os09g0532000), a senescence-inducible NAC transcription factor gene (Os03g0327800), *Lhcb1a* (Os01g0600900), *Lhcb1b* (Os09g0346500), *Actin2* (Os03g0654600), *HemA1* (Os10g0502400), *CAO* (Os10g0567400), and *Lhca4* (Os08g0435900). The following genes of other species were used: *AtLhca4* (AT3G47470), *GmLhca4* (Glyma.04G167900), and *SlLhca4* (Solyc06g069730).

## Results

### Isolation of a rice stay-green mutant, *dye1*

**Fig. 1.**
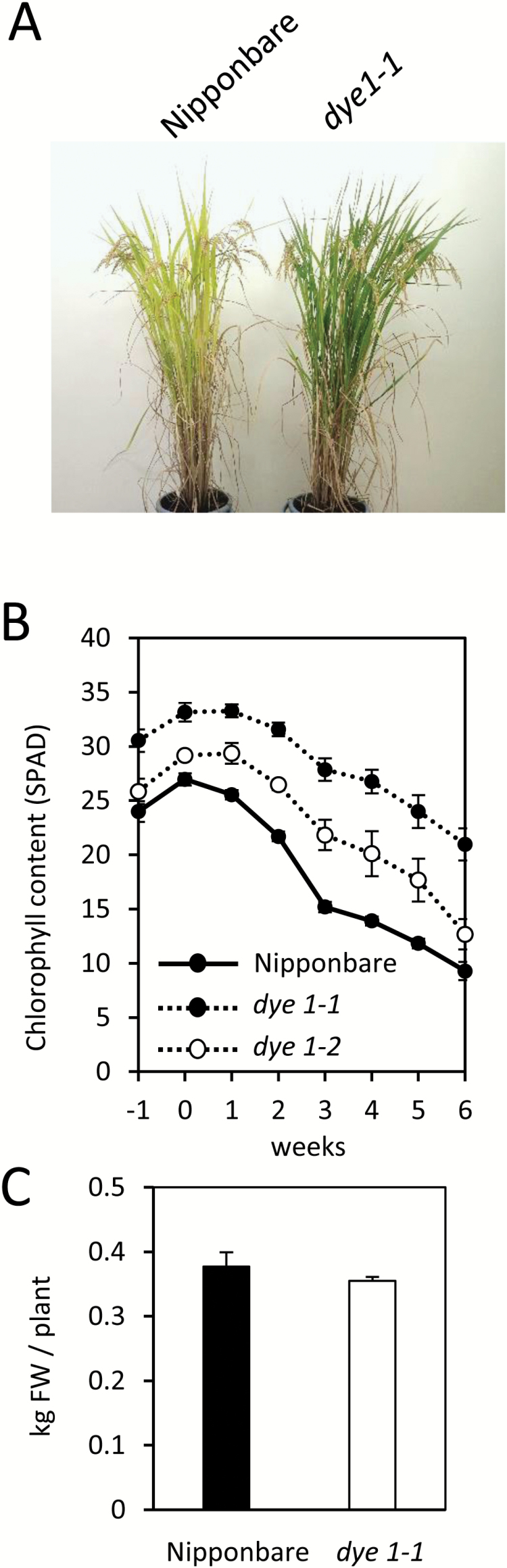
The ‘stay-green’ phenotype of *dye1* during natural leaf senescence. (A) Natural senescence of *dye1-1*, which has greener leaves than wild-type Nipponbare 5 weeks after heading. (B) Changes in the chlorophyll content over time in the flag leaves during natural senescence. The *x*-axis indicates weeks before or after heading. Nipponbare, *dye1-1*, and *dye1-2* are as indicated in the key. Data are means ±SE (n=5). (C) Biomass of *dye1-1* and wild-type Nipponbare at 1 week before heading (means and SE; *n*=5).

In a screening of rice mutants in the field, we isolated one that showed delayed yellowing during natural senescence. This recessive mutant, named *delayed yellowing1-1* (*dye1-1*), was greener than the wild-type cultivar, Nipponbare, 5 weeks after heading, when most leaves are senescent (Fig. 1A). Measurement of the chlorophyll content of flag leaves during the ripening period showed that *dye1-1* had higher chlorophyll contents not only 6 weeks after heading (SPAD units: 9.28 ± 0.83 in Nipponbare, 20.96 ± 1.48 in *dye1-1*) but also 1 week before heading, i.e. before leaf senescence set in (23.98 ± 0.96 in Nipponbare, 30.54 ± 1.04 in *dye1-1*) (Fig. 1B). The shoots of Nipponbare and *dye1-1* plants were harvested 1 week before heading and there was no significant difference between their weights (Fig. 1C).

**Fig. 2.**
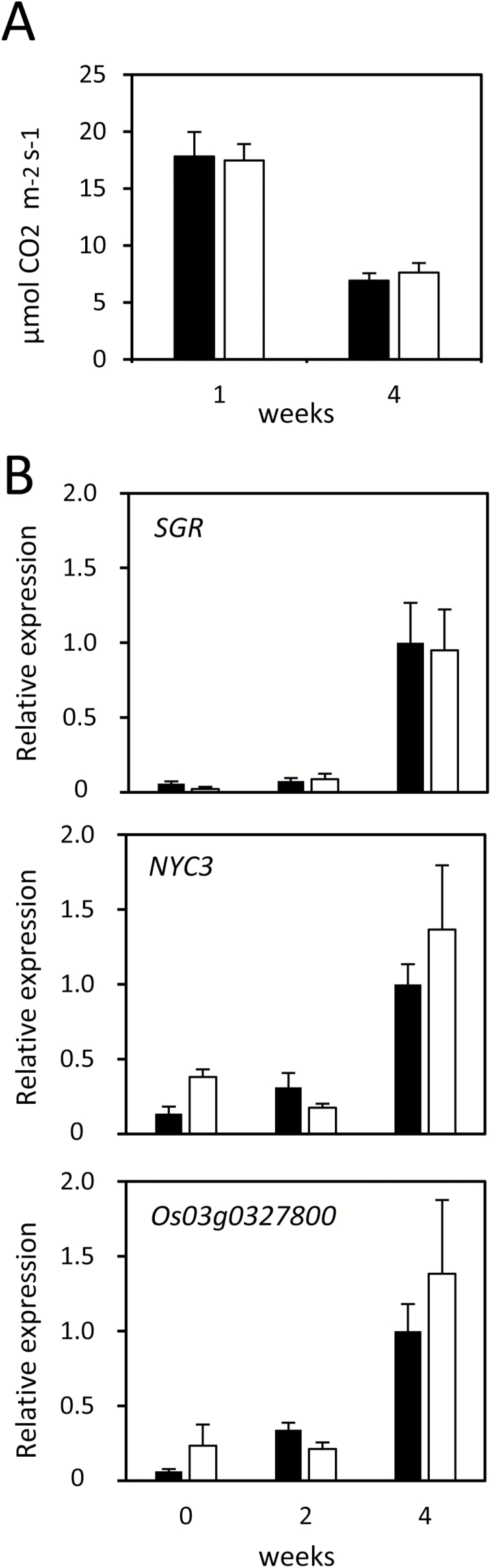
Physiological characterization of *dye1-1* during senescence. (A) Carbon assimilation rates of *dye1-1* (open bars) and wild-type Nipponbare (filled bars) flag leaves 1 week (pre-senescent) and 4 weeks (senescent) after heading. (B) Expression of senescence-associated genes during natural senescence in flag leaves of *dye1-1* (open bars) and Nipponbare (filled bars). Data are means and SE (*n*=4).

Examination of the leaf functionality of *dye1-1* during leaf senescence showed that, in terms of carbon assimilation rate, there was no significant difference between Nipponbare and *dye1-1*, either for pre-senescent leaves (1 week after heading: Nipponbare, 17.81 ± 2.15 μmol CO_2_ m^–2^ s^–1^; *dye1-1*, 17.47 ± 1.43 μmol CO_2_ m^–2^ s^–1^) or for senescent leaves (4 weeks after heading: Nipponbare, 6.94 ± 0.62 μmol CO_2_ m^–2^ s^–1^; *dye1-1*, 7.65 ± 0.82 μmol CO_2_ m^–2^ s^–1^) (Fig. 2A). In addition, we examined the expression of senescence-inducible genes during leaf senescence (Fig. 2B). The expression of the chlorophyll-degrading pathway enzyme-coding genes *SGR* and *NYC3*, as well as Os03g0327800, a senescence-inducible NAC transcription factor gene, were low at heading but were significantly induced 4 weeks after heading in both Nipponbare and *dye1-1*, with no significant differences between them. These observations show that *dye1-1* does not have higher photosynthetic capacity and delayed leaf senescence, despite having greener leaves during senescence.

### Positional cloning of *DYE1*

**Fig. 3.**
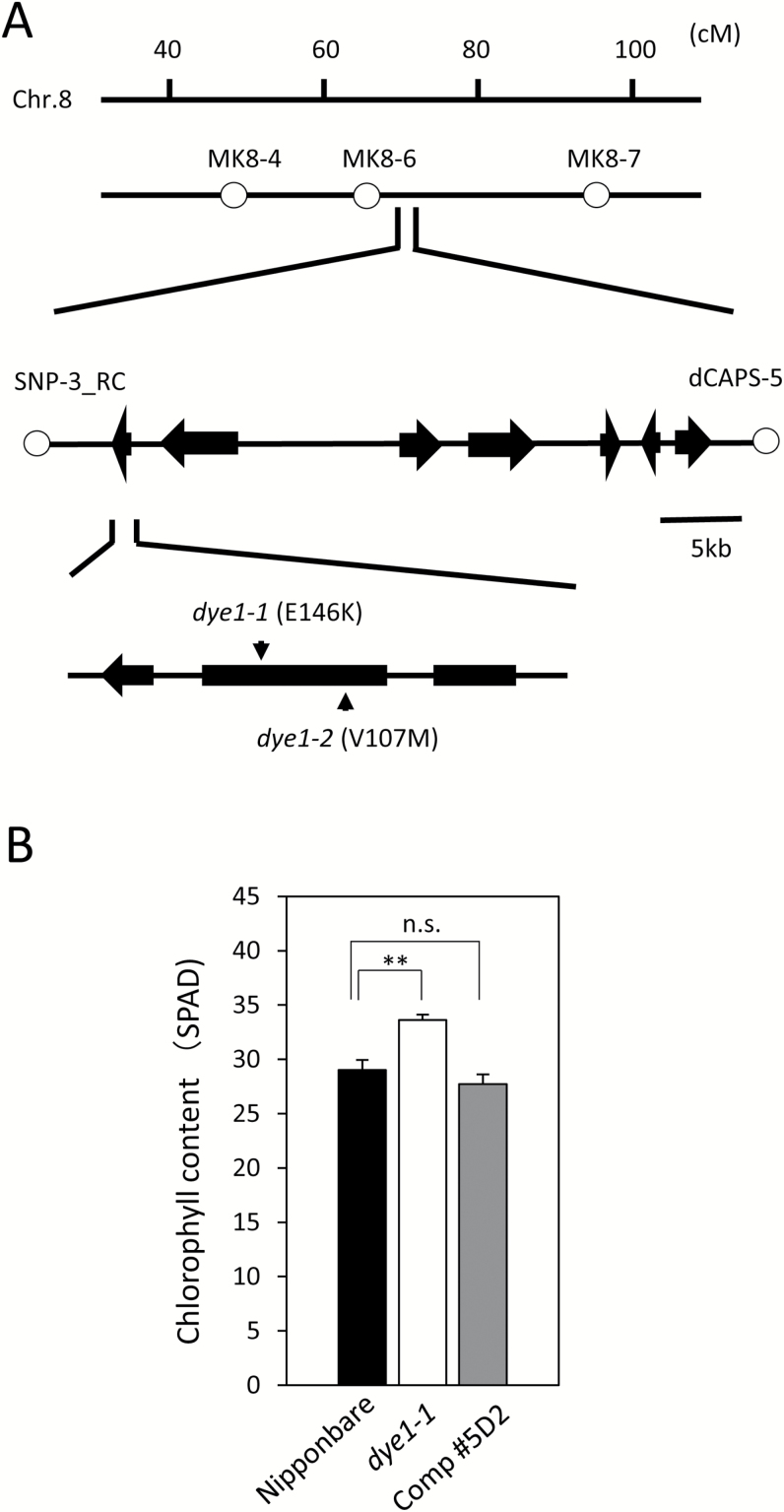
Positional cloning of *DYE1*. (A) Fine-mapping of *DYE1*. Analysis of the F_2_ population and its progeny revealed that *DYE1* is located between the markers SNP-3_RC and dCAPS-5 on Chromosome 8. The open circles indicate makers tightly linked with *DYE1*. *dye1-1* and *dye1-2* have single-nucleotide substitutions causing amino acid substitution in Lhca4. (B) Complementation of *dye1-1* by a *Lhca4* genomic clone. The chlorophyll contents of the flag leaves at heading are shown for wild-type Nipponbare, *dye1-1*, and the complementation transgenic line. Data are means and SE (*n*=4). **P<0.01; n.s., not significant (Student’s *t*-test).

To isolate the *DYE1* gene, we obtained an F_2_ population between *dye1-1* and Gimbozu EG4, a *japonica* strain that harbors over a 1000 copies of *mPing*, a mobile MITE in rice (Nakazaki *et al.*, 2003; Naito *et al.*, 2006). Transposed *mPing*s can be used as sequence characterized amplified region (SCAR) makers for gene mapping (Monden *et al.*, 2009). Coarse-mapping of 22 plants showing the stay-green phenotype selected from this segregating population detected a linkage between *DYE1* and the *mPing*-SCAR marker MK8-6 (77.6 cM) on Chromosome 8 (Fig. 3A). For fine-mapping, we generated an F_2_ population from a cross between *dye1-1* and a CSSL that had its Chromosome 8 replaced with that of the *indica* cultivar Kasalath. Genotyping around 3000 F_2_ plants and their progeny revealed several recombinants near the *DYE1* candidate region (see Supplementary Fig. S1). Analysis of the genotype and phenotype of the recombinants revealed that *DYE1* is located between the derived cleaved-amplified polymorphic sequence (dCAPS) markers SNP-3 _RC and dCAPS5 (Fig. 3A). This 43.1-kb candidate region contains eight functional genes. Whole-genome sequencing of *dye1-1* using Illumina HiSeq2000 revealed a G-to-A substitution in the second exon of *Lhca4* (Os08g0435900), which was the only mutation in the candidate region. This single-base change could cause amino acid substitution from Glu to Lys at position 146 from the first Met in Lhca4, which is a subunit of LHCI. This amino acid residue is highly conserved among the Lhca and Lhcb subunits, and is involved in pigment binding (Melkozernov and Blankenship, 2003). Taken together with the fact that rice has only one copy of the *Lhca4* gene in its genome, it suggests that this amino acid substitution causes a severe impairment of Lhca4 function (see Supplementary Fig. S2) (Melkozernov and Blankenship, 2003; Klimmek *et al.*, 2006). Western blot analysis revealed that the content of the Lhca4 apoprotein was severely reduced in *dye1-1*, suggesting that E146K substitution drastically reduces the stability of the Lhca4 protein (Supplementary Fig. S3).

*dye1-2,* another allele of *dye1*, isolated by TILLING-based screening (Suzuki *et al.*, 2008) of the Nipponbare mutant population, was found to have a single-base change, causing substitution from Val to Met at position 107 from the first Met (Fig. 3A). *dye1-2* showed higher chlorophyll content than Nipponbare 1 week before heading, and the stay-green phenotype during natural senescence, albeit to a weaker degree than *dye1-1* (Fig. 1B).

A complementation experiment was designed to confirm that *DYE1* encodes Lhca4, and was performed via the *Agrobacterium*-mediated transformation method, wherein *dye1-1* was transformed with a 6-kb genomic fragment carrying the entire coding region of the wild-type *Lhca4* gene. This showed that Lhca4 content was significantly reduced in *dye1-1*, but the complementation lines accumulated normal amounts of Lhca4, as expected (see Supplementary Fig. S3). In these complementation lines, the chlorophyll contents of the flag leaves 1 week before heading were similar to those in Nipponbare (Fig. 3B). These results confirmed that *DYE1* encodes Lhca4.

### Photosynthetic properties of *dye1*

**Fig. 4.**
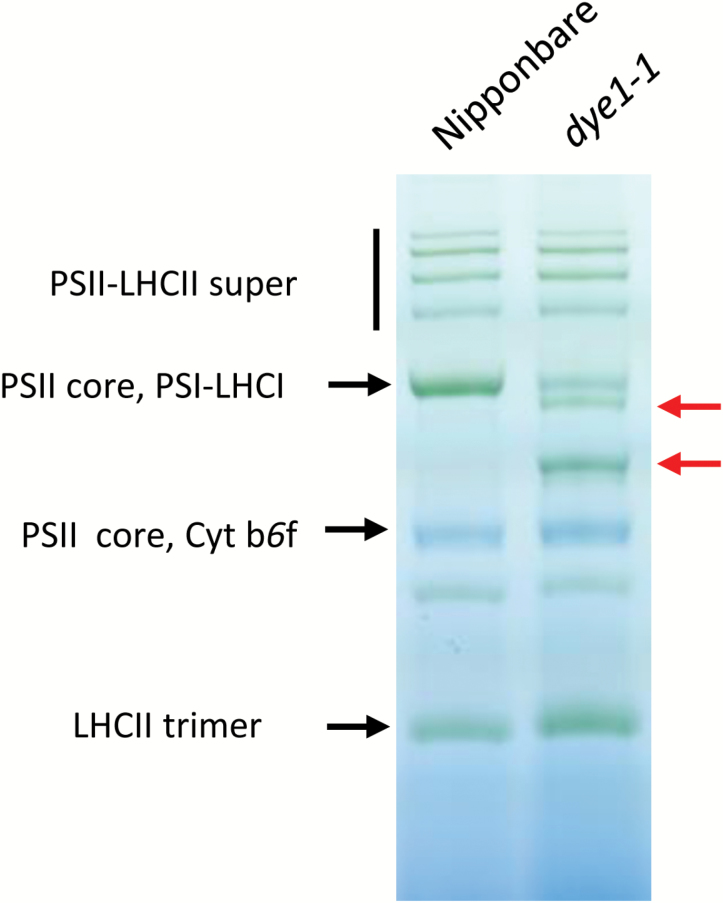
Blue native PAGE analysis of thylakoid proteins obtained from wild-type Nipponbare and *dye1-1* pre-senescent leaves. The thylakoid was solubilized in 1% (w/v) *n*-Dodecyl-β-D-maltoside and subjected to blue native PAGE analysis. Red arrows indicate the newly emerged bands in *dye1-1*. Similar results were obtained across independent experiments.

**Fig. 5.**
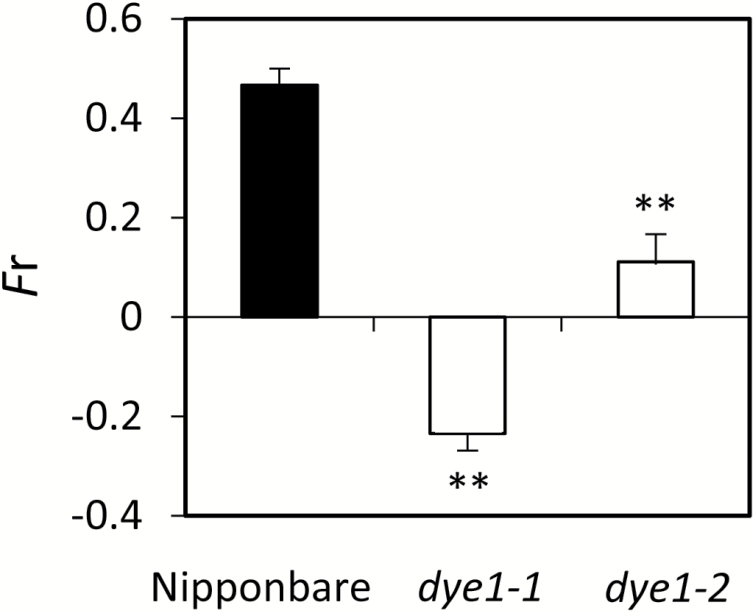
State transition in wild-type Nipponbare, *dye1-1*, and *dye1-2*. *F*_r_ values, which reflect state transition, were measured using mature leaves. Data are means and SE (*n*=3). **P<0.01 (Student’s *t*-test).

We examined the photosynthetic properties of *dye1-1* using flag leaves at heading, since *DYE1* encodes a subunit of LHCI. Both Chl*a* and Chl*b* contents were higher in *dye1-1* than in Nipponbare (1.85 ± 0.09 and 0.57 ± 0.02 nmol mg^–1^ FW for Chl*a* and Chl*b*, respectively, in Nipponbare; 2.66 ± 0.08 and 0.79 ± 0.04 nmol mg^–1^ FW for Chl*a* and Chl*b*, respectively, in *dye1-1*), but the Chl*a*/*b* ratio was similar (see Supplementary Table S3). *F*_v_/*F*_m_ was slightly higher in *dye1-1* than in Nipponbare (Supplementary Table S3). To examine the structure of PSI–LHCI super-complexes, blue native PAGE analysis was performed using mature leaves (Fig. 4). In *dye1-1*, two bands emerged (indicated by red arrows in Fig. 4), which were not observed in Nipponbare. The upper band is thought to correspond with the PSI–LHCI super-complex lacking Lhca4, and the lower band corresponds with the PSI core complex lacking all Lhca subunits. The PSI core complex band was prominent in *dye1-1*, suggesting a severely defective organization of the PSI–LHCI super-complex. Consistent with this observation, the kinetics of P700 oxidation induced by far-red light, which reflects the antenna function of LHCI (Gobets and van Grondelle, 2001; Bonente *et al.*, 2012), was found to be much slower in *dye1-1* (Supplementary Table S3). Interestingly, the *F*_r_ value, an indicator of state transition, was very low in *dye1-1* and moderately low in *dye1-2*, which was consistent with the severity of their phenotype (Fig. 5) (Lunde *et al.*, 2000). A similar observation has been reported in Lhca4 and other Lhca subunit mutants in Arabidopsis (Benson *et al.*, 2015). It is likely that the reduced state transition in *dye1-1* was due to the reduced function of LHCI, and was not specific to Lhca4.

### Analysis of chlorophyll–protein complexes in *dye1*

**Fig. 6.**
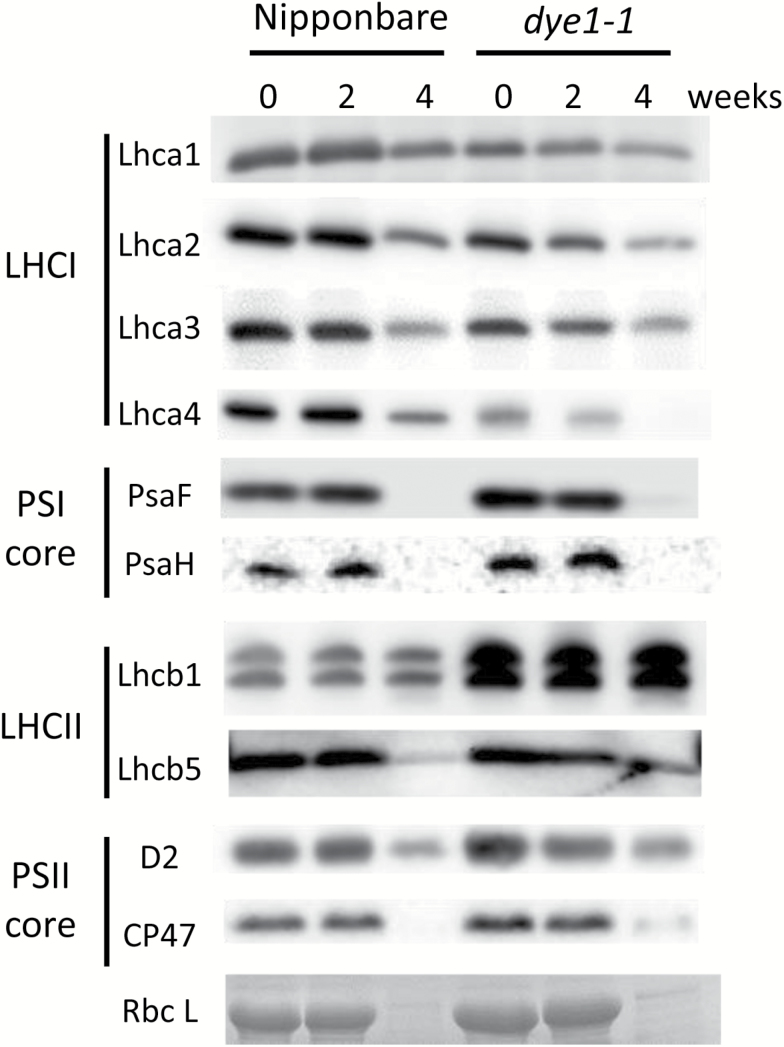
Western blot analysis of photosynthetic proteins in wild-type Nipponbare and *dye1-1* pre-senescent and senescent leaves. Proteins were extracted from the same weight of flag leaves with the same volume of extraction buffer. Similar results were obtained across independent experiments. The Rubisco large subunit was visualized using Coomassie brilliant blue G-250 staining.

The results of western blot analyses, performed for a number of photosynthetic proteins (Fig. 6), showed that the Lhca4 content was severely reduced in pre-senescent (at heading) and senescent (4 weeks after heading) leaves of *dye1-1*. E146K substitution is thought to influence the stability of the Lhca4 protein, as mentioned above. Other Lhca subunits, Lhca1–Lhca3, were also reduced in content in the pre-senescent and senescent leaves of *dye1-1*. It is very likely that the impairment of Lhca4 destabilizes other Lhca subunits because of defects in the proper formation of the PSI–LHCI super-complex. Slight increases in the subunits of the PSII core, D2 (1.4-fold) and CP47 (1.3-fold), were observed in the *dye1-1* flag leaves at heading, suggesting that the amount of PSII core complex increased slightly. In addition, a slight increase in the PSI core subunits PsaF (1.5-fold) and PsaH (1.5-fold) were observed in the *dye1-1* flag leaves at heading, suggesting a slight increase in the PSI core complex. Interestingly, a more prominent increase was observed in a trimeric LHCII subunit in *dye1-1*. The content of Lhcb1 in the flag leaves at heading in *dye1-1* was 2.6-fold that of the equivalent leaves in Nipponbare. In contrast, there was no significant increase in the content of Lhcb5, a monomeric LHCII subunit, in *dye1-1*. Levels of several chlorophyll–protein complexes were elevated in *dye1-1*, suggesting that the higher chlorophyll content in the pre-senescent *dye1-1* leaves was due to an increase in such complexes, particularly trimeric LHCII.

**Fig. 7.**
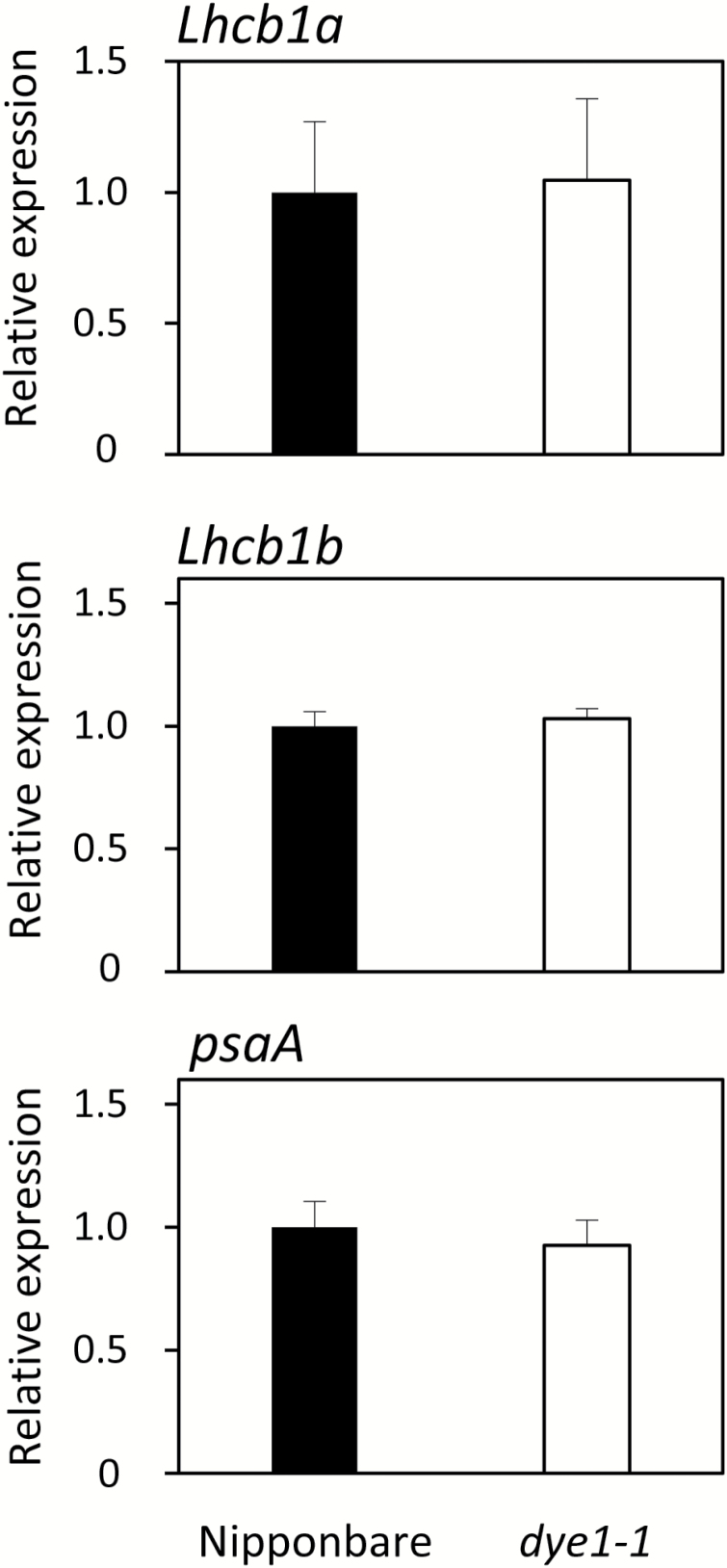
Expression of *Lhcb1* and *psaA* in wild-type Nipponbare and *dye1-1*. Quantitative RT-PCR analysis of *Lhcb1a*, *Lhcb1b*, and *psaA* was performed using flag leaves at heading. Data are means and SE (*n*=4).

In rice, there are two copies of the *Lhcb1* gene, *Lhcb1a* and *Lhcb1b*. qPCR analysis revealed no significant up-regulation of *Lhcb1a* and *Lhcb1b* expression in *dye1-1*, suggesting that the increase in Lhcb1 protein in *dye1-1* was not regulated at the mRNA level (Fig.7). Similarly, there was no increase in the mRNA of the PSI core subunit PsaA in *dye1-1*. As it is known that LHCII accumulation is regulated by chlorophyll content, particularly Chl*b*, the expression of the genes involved in chlorophyll synthesis was investigated. The mRNA level of *HemA1*, which encodes the rate-limiting enzyme of tetrapyrrole synthesis, Glu-tRNA reductase, was not significantly different between Nipponbare and *dye1-1* (Tanaka *et al.*, 2011) (see Supplementary Fig. S4). Similarly, the mRNA level of *CAO*, which encodes the Chl*b*-synthesizing enzyme chlorophyllide *a* oxygenase, was comparable between Nipponbare and *dye1-1* (Supplementary Fig. S4).

## Discussion

*dye1* was isolated as a delayed-yellowing mutant of rice in a field experiment. The pre-senescent mature leaves of *dye1* contained a higher level of chlorophyll compared with those of the wild-type, suggesting that this elevated chlorophyll content causes the ‘stay-green’ phenotype of the senescent leaves. However, *dye1* showed comparable levels of CO_2_ assimilation rate and expression of senescence-inducible genes in the senescent leaves, suggesting that it is a non-functional stay-green mutant. Taken together, the results suggest that *dye1* is a Type E stay-green mutant (Thomas and Howarth, 2000). To our knowledge, this is the first empirical report on such a mutant.

Positional cloning revealed that *DYE1* encodes Lhca4, a subunit of the PSI antenna complex LHCI. While *dye1* is the only *Lhca* mutant reported in rice so far, two *Lhca* mutants have been reported in Arabidopsis, including a quadruple-mutant of Lhca1–Lhca4 (ΔLhca) (Ganeteg *et al.*, 2004; Benson *et al.*, 2015; Bressan *et al.*, 2016). These mutants are reported to show reduced state transition, and slightly higher *F*_v_/*F*_m_ values and Chl*a*/*b* ratios (Benson *et al.*, 2015; Bressan *et al.*, 2016), which are common to *dye1-1*. In addition to these characteristics, *dye1-1* showed higher chlorophyll content, which has not been described in the *Lhca* mutants in Arabidopsis. The fact that *dye1-1*, a mutant of a chlorophyll–protein complex, has a higher chlorophyll content than the wild-type is thought to be due to a higher accumulation of other chlorophyll–protein complexes, particularly the trimeric LHCII.

*dye1-1* has an E146K amino acid substitution in Lhca4. This residue corresponds to E154 in Arabidopsis Lhca4, which is a pigment-binding site conserved not only among the Lhca subunits, but also among the Lhcb subunits of different species (Melkozernov and Blankenship, 2003). Furthermore, western blot analysis revealed that the Lhca4 apoprotein content was severely reduced in *dye1-1*. Taken together, these results suggest a severe impairment of Lhca4 function in *dye1-1*. Interestingly, the phenotype of the *Lhca4* mutant is the severest among the single Lhca-subunit mutants in Arabidopsis (Benson *et al.*, 2015). This is partly because the impairment of Lhca4 drastically influences the stability of other LHCI subunits and the organization of the PSI–LHCI super-complex. Indeed, the kinetics of P700 oxidation by far-red light was much slower in *dye1-1*. Nevertheless, the biomass and CO_2_ assimilation efficiency in *dye1-1* were comparable with those of the Nipponbare pre-senescent leaves.

Plants adapt to an imbalance in PSI/PSII through short- and long-term acclimations. In Arabidopsis, long-time exposure of PSII-activating light dramatically increases the transcription of *psaA*/*pasB* and the accumulation of PSI core proteins (Pesaresi *et al.*, 2009). Because the LHCI antenna was reduced in *dye1*, the activity of PSI is thought to be continuously low. However, only a slight increase in the PSI core and no increase in *psaA* transcript accumulation were observed in *dye1-1*, suggesting that the mechanism of long term-acclimation in rice is different from that in Arabidopsis. In contrast, higher LHCII accumulation was observed in *dye1-1*. It is possible that the increase in trimeric LHCII accumulation is a kind of compensation for a reduced PSI antenna. LHCII has been classically thought to be the antenna of PSII, but increasing evidence suggests that it could be a major antenna of PSI, even under normal conditions. Indeed, Bressan *et al.* (2016) suggested that LHCII can compensate for the deficiency of LHCI, although not perfectly.

The mRNA levels of *Lhcb1a* and *Lhcb1b* did not increase in *dye1-1*, suggesting that the increase in Lhcb protein was regulated not at the mRNA level, but at the protein level. For instance, it is suggested that the *gun*-related retrograde signaling, which regulates *Lhcb* transcription, is not involved in this phenomenon (Kleine and Leister, 2016). Lhcb protein accumulation is known to be regulated by chlorophyll content, particularly by Chl*b* (Bellemare *et al.*, 1982; Horn and Paulsen, 2004; Kusaba *et al.*, 2007); however, expression of the enzyme genes *HemA1* and *CAO* involved in chlorophyll synthesis did not differ significantly between Nippponbare and *dye1-1*, even though these enzymes are also under post-translational control (Nakagawara *et al.*, 2007; Tanaka *et al.*, 2011). Thus, the detailed mechanism behind the increase in LHCII content in *dye1-1* remains unclear. An increase in the LHCII content in *dye1-1* might be a compensation for the low activity of PSI, considering that the biomass and carbon assimilation rate of *dye1-1* were comparable to those of the wild-type, despite very low antenna function of LHCI. Consistent with this idea, Lhca4-antisense plants in Arabidopsis show considerable growth defects under field and controlled conditions (Ganeteg *et al.*, 2004). However, the possible involvement of reduced state transition cannot be excluded. In any case, our observation that the impairment of Lhca4 caused increased LHCII accumulation suggests that LHCI and LHCII functionally interact in a previously unknown manner.

## Supplementary data

Supplementary data are available at *JXB* online.

Fig. S1. Genotype and phonotype of recombinant individuals in the *DYE1* candidate region.

Fig. S2. Alignment of Lhca4 proteins from different species.

Fig. S3. Western blot analysis of Lhca4 in the complementation line.

Fig. S4. Expression of *HemA1* and *CAO* in *dye1-1*.

Table 1. Primers used in the quantitative RT-PCR

Table 2. Information on dCAPS markers used in the positional cloning of *DYE1*.

Table 3. Analysis of the photosynthetic properties of the pre-senescent flag leaves in *dye1-1*.

## Supplementary Material

Supplementary Figures and Tables

## Abbreviations:

CSSL: Chromosome segment substitution line
dCAPS: derived cleaved-amplified polymorphic sequence
dye1: *delayed yellowing1*
LHC: light-harvesting complex
MITE: miniature inverted–repeat transposable element
SCAR: sequence characterized amplified region.
