# Supplementary material for: Impairment of Lhca4, a subunit of LHCI, causes high accumulation of chlorophyll and the stay-green phenotype in rice
Source: J Exp Bot. 2018 Jan 3;69(5):1027–35. doi: 10.1093/jxb/erx468 (PMC6019047; doi:10.1093/jxb/erx468)
Supplement: Supplementary Figures and Tables [file erx468_suppl_supplementary_figures_and_tables.pdf]

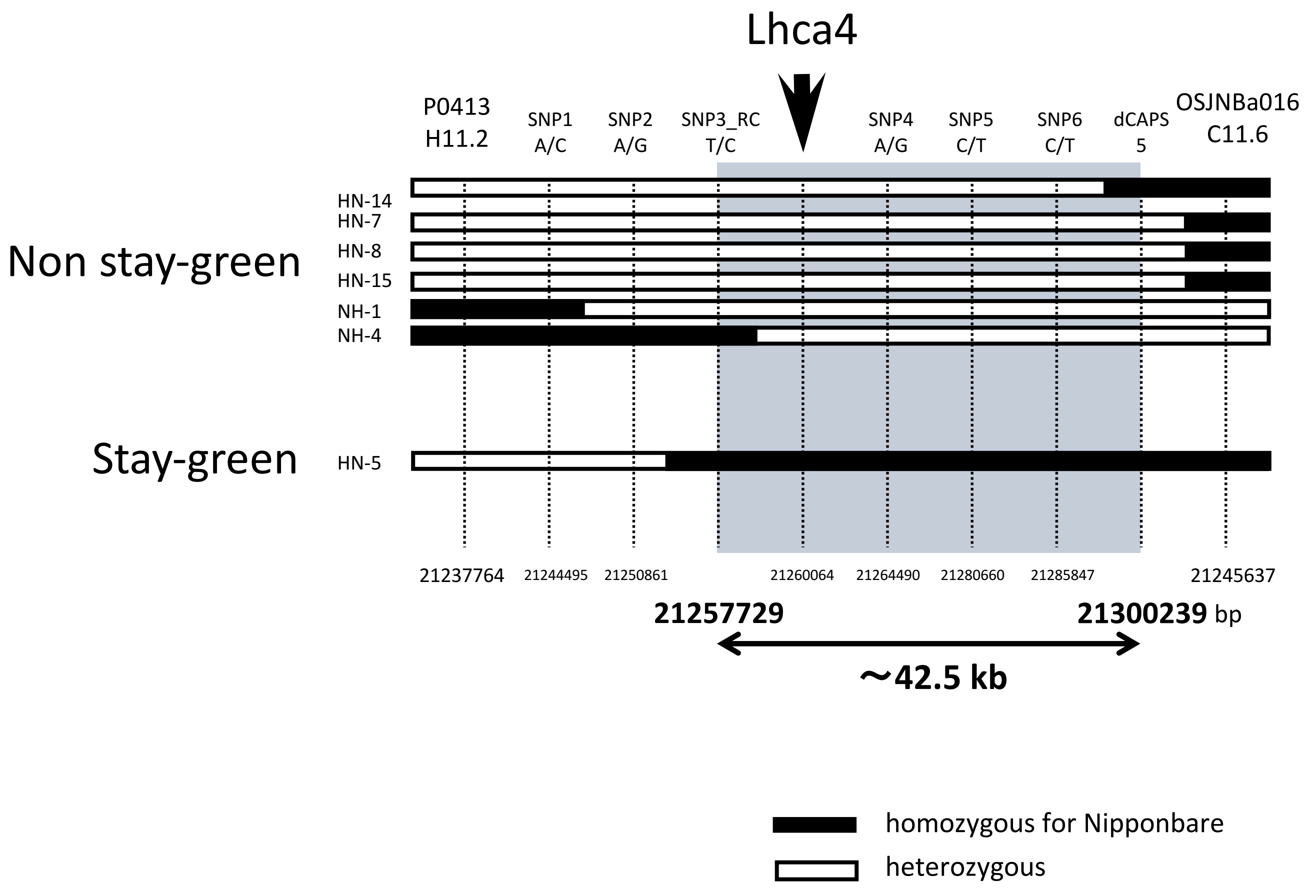

Supplementary Fig. S1. Genotype and phenotype of recombinant individuals in the *DYE1* candidate region. Recombinants obtained from F_2 and its progeny population between *dye1-1* and the CSSL SL224 are shown. The *DYE1* candidate region delimited by these recombinants is shown in gray. SNP positions were referenced by IRGSP Releases Build 5.0.

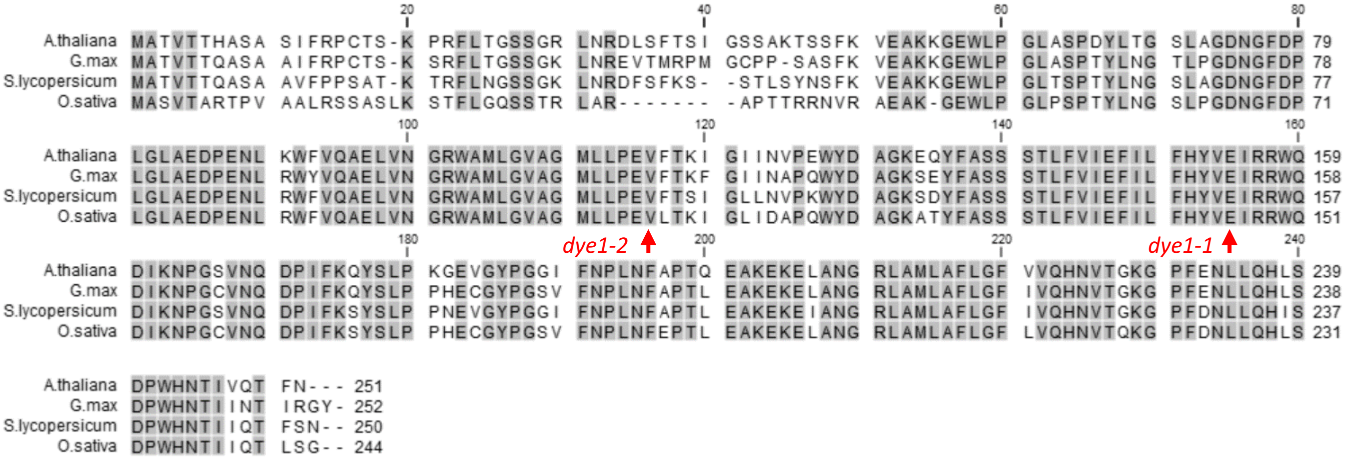

Supplementary Fig. S2. Alignment of Lhca4 proteins from different species.
Conserved amino acid residues are shaded. The substituted amino acid in *dye1-1* (E146K) and *dye1-2* (V107M) are shown with arrows.

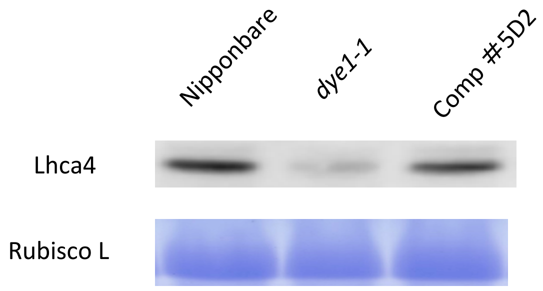

Supplementary Fig. S3. Western blot analysis of Lhca4 in the complementation line.
Lhca4 was detected by western blot analysis in *dye1-1* and the complementation transgenic line Comp #5D2. Rubisco L was visualized by Coomassie brilliant blue G-250 staining.

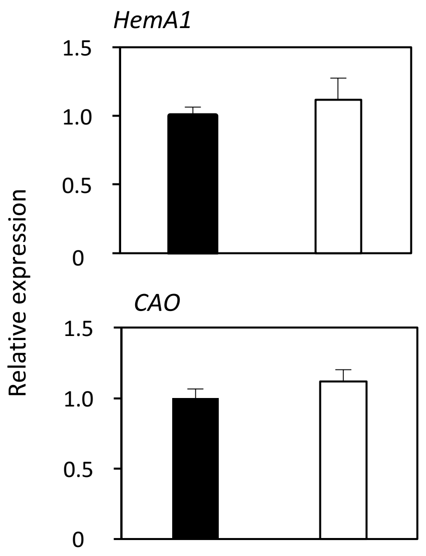

Supplemental Figure S4. Expression of *HemA1* and *CAO* in *dye1-1*. Quantitative RT-PCR analysis of expression of *HemA1* and *CAO* in flag leaves of 1 week before heading. Filled and open bars show Nipponbare and *dye1-1*, respectively. Thin bars indicate standard errors (n = 4).

Supplementary Table S1. Primers used in the quantitative RT-PCR.

Gene	Forward primer	Reverse primer
Lhcb1a	AGTAGCTGAGCTTGAAGCAG	GAACTCGCCGGTCAGGTAG
Lhcb1b	GGAGCTCAAGGTGAAGGAGA	GTCCCCCACTTCACTTGCCG
PsaA	ATTAGCCTGGAGTCATGACG	TTCATGAGGAAGTGGTATCTCC
SGR	CGCTACTACATCTTCCGCAA	TGGAGTGGAAGTAGACCCACA
NYC3	TCTATCTAGGTGCCAAAGGC	ATTCTGGCACCTGCTGTTTC
Os03g0327800	AAGCGCAAGAGATCATCGGA	CTCAGTCCCAGTGACGATCC
HemA1	GTCAAAGAAAAGTGGAATCCC	AGGATTTGCCCTTCTCCAAG
CAO	TCACGCAGAGATAGTGATGG	AATTCCATGTCGATCGGGTA
actin2	TTCCAACAGATGTGGATCTCA	GGACACCAACAATCCCAAAC

Supplementary Table S2. Information for dCAPS markers used in the positional cloning of *DYE1*

Name	Reverse primer	Reverse primer	Restriction enzyme
P0413H11.2	ATGCCACATGACTGAGTACTGCAGCCAAGA	TTCAGATTACATGGCGCTGC	SacI
Lhca4	ATGTCCTGCCACCGCCGGAGCT	CAGGCGATAACGGGTTCGAC	SacI
dCAPS_5	GCTTGTGTCAGTTGTGT	ATGTATGAAATAATCAAAGATAACATTA	MseI
OSJNBa0016C11.6	TCTCCCTATCGAGATGGCGA	TCGATCTTGGACAAGGGGAGCTCCTCCTTC	XbaI

Supplementary Table S3. Analysis of photosynthetic properties of presenescent flag leaves in *dye1-1*.

	Chl*a* mmol /mg FwW	Chl*b* mmol /mg FwW	Total Chl mmol /mg FW	Chl*a*/*b*	Fv/Fm	Maximum P700+ 2/3 T sec
Nipponbare	1.85±0.09	0.57±0.02	2.42±0.11	3.21±0.08	0.82±0.001	9.60±2.02
dye1-1	2.66±0.08 **	0.79±0.04 **	3.43±0.12 **	3.38±0.10	0.86±0.063 **	62.69±13.91 **

Flag leaves at heading were used for analysis. Maximum P700+ 2/3 T: Time to reach 2/3 of maximum P700+ (sec). n=4 for Chla, Chlb, Total Chl, Chla/b. n=5 for Maximum P700+ 2/3 T. **, P<0.01 (Student's t-test)
